# The relative and interactive effects of urinary multiple metals exposure on hyperuricemia among urban elderly in China

**DOI:** 10.3389/fpubh.2023.1015202

**Published:** 2023-02-13

**Authors:** Chao Huang, Erwei Gao, Feng Xiao, Qiongzhen Wu, Wei Liu, Yi Luo, Xiaohu Ren, Xiao Chen, Kaiwu He, Haiyan Huang, Qian Sun, Desheng Wu, Jianjun Liu

**Affiliations:** ^1^Shenzhen Key Laboratory of Modern Toxicology, Shenzhen Medical Key Discipline of Health Toxicology (2020–2024), Shenzhen Center for Disease Control and Prevention, Shenzhen, Guangdong, China; ^2^Food Inspection and Quarantine Technology Center of Shenzhen Customs, Shenzhen, Guangdong, China; ^3^Shenzhen Luohu Hospital for Traditional Chinese Medicine, Shenzhen Luohu Hospital Group, Shenzhen, Guangdong, China

**Keywords:** urinary metal exposure, hyperuricemia, serum uric acid, interactions, dose-response relationship

## Abstract

**Objective:**

Independent and interactive effects of multiple metals levels in urine on the risk of hyperuricemia (HUA) in the elderly were investigated.

**Methods:**

A total of 6,508 individuals from the baseline population of the Shenzhen aging-related disorder cohort were included in this study. We detected urinary concentrations of 24 metals using inductively coupled plasma mass spectrometry, fitted unconditional logistic regression models, and the least absolute shrinkage and selection operator regression models for the selection of metals as well as unconditional stepwise logistic regression models and restricted cubic spline logistic regression models for assessing the associations of urinary metals and HUA risk, and finally applied generalized linear models to determine the interaction with urinary metals on the risk of HUA.

**Results:**

Unconditional stepwise logistic regression models showed the association between urinary vanadium, iron, nickel, zinc, or arsenic and HUA risk (all *P* < 0.05). We revealed a negative linear dose–response relationship between urinary iron levels and HUA risk (*P*_*overall*_ < 0.001, *P*_*nonliner*_ = 0.682), a positive linear dose–response relationship between urinary zinc levels and HUA risk (*P*_*overall*_ < 0.001, *P*_*nonliner*_ = 0.513), and an additive interaction relationship between urinary low-iron and high-zinc levels and HUA risk (RERI = 0.31, 95% CI: 0.03–0.59; AP = 0.18, 95%CI: 0.02–0.34; S = 1.76, 95%CI: 1.69–3.49).

**Conclusion:**

Urinary vanadium, iron, nickel, zinc, or arsenic levels were associated with HUA risk, and the additive interaction of low-iron (<78.56 μg/L) and high-zinc (≥385.39 μg/L) levels may lead to a higher risk of HUA.

## Introduction

Hyperuricemia (HUA) is the second most common metabolic disease in China; it has been a concern because of its association with cardiovascular diseases and chronic kidney diseases (1–3). HUA is defined as a serum uric acid (SUA) level of ≥ 7.0 mg/dl for men and ≥ 6.0 mg/dl for women. A meta-analysis of 59 studies on HUA in China during 1995–2010 suggested that the crude prevalence of HUA in the Chinese population was 13.3, 19.4% for men and 7.9% for women (3), and the age-standardized prevalence of HUA in the Chinese population increased with age from 9.5% in individuals aged 60–64 years to 21.9% in those aged 80+ years (4). Individuals with higher SUA were at high risk for hypertension, metabolic syndrome, acute myocardial infarction, and Alzheimer's disease (5–8). Moreover, a relationship between SUA levels and traditional risk factors including gender, age, dietary habit, and body mass index (BMI) was found (9); however, limited evidence of non-traditional risk factors for elevated serum SUA levels is available in the literature.

Multiple metals can enter the body through the inhalation of air, tobacco smoking, ingestion of drinking water and food, and skin contact, which may induce pathological responses as well as many diseases, such as cardiovascular disease, cancer, and kidney disease (10–13). Evidence indicated that environmental exposure to metals is related to HUA in American and Chinese adults (14–17) and multiple metals exposure can cause metabolic disorders and cognitive impairment in elderly Chinese persons (18, 19). Based on the data from the National Health and Nutrition Examination Survey (NHANES) during the period from 2003 to 2010, Kuo et al. found that HUA risk was 1.84 times higher in men with total urinary arsenic (As) of ≤ 4.2 μg/L than those with total urinary As of >17.3 μg/L (14). A recent cross-sectional study in Shenzhen, China regarding routine physical examination data (*n* = 1,406, ranging in age from 31 to 91 years) suggested a positive dose–response relationship between plasma levels of zinc (Zn) or As and HUA risk (15). Several studies in Changsha, China showed that among 6,212 adults aged above 40 years, serum copper (Cu) levels showed a positive relationship with HUA risk after adjusting for potential confounders (age, gender, BMI, smoking, drinking, education, occupation, hypertension, and diabetes) (16), and among 2,120 adults aged 20–75 years, only women with total blood lead (Pb, >126 μg/L) had a 2.19-fold higher risk of HUA (17). Nevertheless, literature regarding the adverse effects of multiple metals co-exposure on HUA is limited.

Certain metals could show synergistic and antagonistic interactions with other metals on human health by promoting or inhibiting the absorption of other metals (20). The NHANES (2011–2016) study revealed that whole-blood Pb showed a synergistic interaction with blood manganese on the reduced bone mineral density (21). A recent study (*n* = 2,882 individuals with a mean age of 65.58 years) from the Dongfeng-Tongji cohort indicated that higher plasma concentrations of selenium (Se) with Zn decreased the positive association between plasma Cu and C-reactive protein (22). However, many studies on plasma or serum levels of metals indicated the differences in metal concentrations in various biological samples and their biological significance. For example, urinary cadmium (Cd) concentrations reflect a long-term accumulation of Cd in the kidneys (23), while blood Cd concentrations mainly reflect recent exposure to Cd (24). Therefore, the relationship between metal concentrations in humans and HUA risk cannot be fully explained by blood metal concentrations. We measured urinary metal concentrations (25) and SUA levels to explore the association between urinary levels of multiple metals with hyperuricemia risk in elderly residents in Shenzhen.

## Materials and methods

### Subjects

This cross-sectional study is based on the baseline data from the Shenzhen aging-related disorder cohort (26). The baseline population consisted of 9,411 elderly residents (≥ 60 years) with a Shenzhen household registration from the 51 community rehabilitation centers in a district of Shenzhen by random cluster sampling methods, during the period from July 2017 to November 2018. They participated in a health questionnaire and physical examination. To investigate the association between urinary metals levels and HUA risk, we first excluded 36 individuals with self-reported kidney diseases and 1,022 individuals with an estimated glomerular filtration rate (eGFR) of <60 ml/min per 1.73 m^2^ from the baseline population. Thereafter, we excluded 1,845 individuals who missed data on educational information (*n* = 75), marital status (*n* = 123), passive smoking status (*n* = 71), drinking status (*n* = 16), hypertension (*n* = 14), hyperlipidemia (*n* = 14), diabetes (*n* = 23), kidney diseases (*n* = 10), BMI (*n* = 88), and serum creatinine (*n* = 21), as well as 1,390 individuals without urinary metal values. Finally, 6,508 individuals were included in this study. The research protocol was approved by the Medical Ethics Research Committee of Shenzhen Center for Disease Control and Prevention (approval numbers: R2017001 and R2018020). Each participant signed an informed consent form before engaging in the study.

### Data collection

We collected data on the health questionnaire from the participants through the trained investigators in face-to-face interviews. The health questionnaire contained the following items: general demographics, personal and family health histories, tobacco smoking, and alcohol drinking. In this study, HUA was defined as SUA of >420 μmol/L in male participants and >360 μmol/L in female participants (27). Current smoking was defined as those who smoked at least one cigarette per day for more than 6 months; quit smoking was defined as those who had quit smoking at the time of the survey; the rest were considered as never smoking. Current drinking was defined as those who drank at least once a week for over 6 months; quit drinking was defined as those who had quit drinking at the time of the survey; the rest were considered never drinking. Hypertension was defined as systolic blood pressure (SBP) of ≥140 mmHg, diastolic blood pressure (DBP) of ≥90 mmHg, previously diagnosed patients with hypertension patients, or antihypertensive drug use. Diabetes was defined as fasting blood glucose concentration of ≥7.0 mmol/L, previously diagnosed diabetes, or hypoglycemic drug use. Hyperlipidemia was defined as total cholesterol of ≥5.18 mmol/L, triglyceride of ≥1.7 mmol/L, low-density lipoprotein cholesterol of ≥3.37 mmol/L, high-density lipoprotein cholesterol of ≤ 1.0 mmol/L, or previously diagnosed hyperlipidemia or lipid-lowering drug use. eGFR was calculated according to the formula recommended by the Chronic Kidney Disease Epidemiology Collaboration (CKD-EPI) (2009 edition) (28): serum creatinine (Scr) ≤ 0.7 mg/dL, eGFR = 144 × (Scr/0.7)^−0.329^ × (0.993) ^age^; Scr > 0.7 mg/dL, eGFR = 144 × (Scr/0.7)^−1.209^ × (0.993) ^age^ for female participants; Scr ≤ 0.9 mg/dL, eGFR = 141 × (Scr/0.9)^−0.411^ × (0.993) ^age^; Scr > 0.9 mg/dL, eGFR = 141 × (Scr/0.9)^−1.209^ × (0.993) ^age^ for male participants. Data were expressed in ml/min per 1.73 m^2^.

### Urinary metal concentrations

We measured 24 urinary metals in urine samples, including lithium, beryllium (Be), aluminum (Al), titanium, vanadium (V), chromium (Cr), manganese, iron (Fe), cobalt, nickel (Ni), Cu, Zn, As, Se, rubidium, strontium, molybdenum (Mo), Cd, indium (In), tin, antimony (Sb), barium, thallium (Tl), and Pb using inductively coupled plasma-mass spectrometry (ICP-MS, NEXION 300X. PerkinElmer Inc. Waltham, Massachusetts, USA). Briefly, urine samples were thawed at room temperature and then centrifuged (4,200 rpm × 10 min) at room temperature. Afterward, 0.5 ml of supernatant from each urine sample was added into a 15 ml polypropylene tube, and then 4.5 ml of 2% nitric acid solution (10 times the diluted urine sample) was added. To assess the accuracy of the measurements, Seronom^TM^ Trace Elements Urine L-1 (Sero Incorporated Company. Billingstad, Norway), Seronom^TM^ Trace Elements Urine L-2 (Sero Incorporated Company. Billingstad, Norway), and Trace Elements in Natural Water (SRM1640a) (National Institute of Standards and Technology. Gaithersburg, Maryland, USA) were added as quality control samples for each batch. As shown in Supplementary Table 1, the range values of 66.04–152.86% in urinary metals were considered acceptable for spike recoveries. The limits of detection (LOD) ranged from 0 to 1.66 μg/L for urinary Fe and from 0 to 0.13 μg/L for urinary Zn. The limits of quantification (LOQ) ranged from 0.01 to 5.54 μg/L for urinary Fe and from 0 to 0.44 μg/L for urinary Zn. Urinary concentrations of Be, In, and Sb were excluded from further analysis because the values of Be, In, and Sb in more than 80% of individuals were below the corresponding LOD. Values of urinary metals below the LOQ were replaced by LOQ/2.

### Statistical analysis

A Student's *t*-test, Mann-Whitney *U*-test, and Chi-square test were correspondingly used to compare normally, non-normally continuous (including eGFR, urine creatinine, and urinary metals concentrations), and categorical variables (including age, gender, education level, marital status, active smoking status, passive smoking status, drinking status, hypertension, diabetes, hyperlipidemia, and BMI) between the non-hyperuricemia and hyperuricemia groups. Values of urinary metals were log10-transformed before analysis to approximately normal distributions. Values of Spearman's rank correlations coefficient were calculated among the 21 urinary metal concentrations.

We identified individual urinary metals by unconditional logistic regression models or LASSO regression models for further analysis. When constructing unconditional logistic regression models, the participants were divided into four subgroups (i.e., ≤ P25 as the reference group, P25, P50, and P75) according to the quartile values of urinary concentrations of individual metal, after adjusting for potential confounders, including age (<67 or ≥67 years old), gender (male or female), education level (<9, ≥9, or ≥13 years), marital status (married or other marital status), active smoking status (never, quit, or current), passive smoking status (yes or no), drinking status (never, quit, or current), hypertension (yes or no), diabetes (yes or no), hyperlipidemia (yes or no), BMI (<24 or ≥24 kg/m^2^), estimated glomerular filtration rate (eGFR: mL/min per 1.73 m^2^), and urine creatinine (μmol/L). The median value in each metal quartile (log10-transformed urinary metal value) was entered into the logistic regression model as a continuous variable. In the LASSO regression model, 10-fold cross-validation was used to select metals based on the lambda (λ) parameter with minimum mean square error (minimum MSE). Identified metals by both logistical regression models and LASSO regression were included in unconditional stepwise logistic regression models (enter = 0.05 and remove = 0.10). Herein, we adjusted for the same potential confounders in both logistical regression models and LASSO regression. RCS logistic regression models were constructed to analyze the dose–response relationship between urinary metals levels and HUA risk. The knots were set to the 10th, 50th, and 90th percentiles of each metal value, and the 25th percentiles of each metal were set as the corresponding reference value. We also used multiple linear regression models to evaluate the associations between urinary metals and SUA levels.

Generalized linear model (GLM) was used to evaluate the additive interaction of urinary metals on the risk of HUA. Individuals were classified into high (≥ median) and low (<median) subgroups based on urinary metals values. Relative excess risk due to interaction (RERI), attributable proportion due to interaction (AP), and synergy index (S) were used to assess the additive interaction between urinary metals concentrations and HUA risk (29). A regression tree is a machine-learning algorithm known to detect multiple interactions between covariates (30). We used a regression tree to explore multiple interactions between urinary metals and SUA levels.

Subgroup analyses of age, gender, BMI, hypertension, diabetes, or hyperlipidemia were conducted. Individuals were divided into high- and low-metal subgroups according to urinary median values (log10-transformed) of urinary metals. The interaction was examined by adding an interaction term between a specific metal and the stratification variable and adjusted for the same confounders in unconditional logistic regression models. All data were analyzed using Statistical Program for Social Sciences 17.0 (SPSS Inc., Chicago, Illinois, USA). LASSO regression analysis was performed with R 4.2 (Lucent Technologies, USA) “glmnet” package. RCS analysis was conducted using SAS 9.2 (SAS Institute Inc., Cary, North Carolina, USA) (RCS_Reg macro) (31). Statistical significance was defined as a *P-*value of < 0.05 (two-tailed).

## Results

### Participants characteristics

As shown in Table 1, among the 6,508 participants, 2,731 were men and 3,777 were women, and 4,147 were in the non-HUA subgroup and 2,361 were in the HUA subgroup. When compared with individuals in the non-HUA subgroup, those in the HUA subgroup had lower urinary levels of lithium, V, Cr, Fe, Ni, strontium, or Mo as well as higher urinary levels of Zn or As (all *P* < 0.05). As shown in Supplementary Figure 1, Spearman correlation analysis revealed the correlations among 21 metals with each other (all *P* < 0.05), wherein Se showed a strong correlation with titanium, Zn, As, rubidium, and Mo (the corresponding correlation coefficients: 0.76, 0.71, 0.76, 0.74, and 0.74, all *P* < 0.05). However, the correlation of V with Al was weak (correlation coefficient: 0.13, *P* < 0.05).

**Table 1:** Characteristics and urinary metal concentrations of the study population.

**Variable**	**Non-hyperuricemia**	**Hyperuricemia**	***p*-value**
Age (<67/≥67 years, *n* %)	2,149/1,998 (51.8/48.2)	1,180/1,181 (50.0/50.0)	>0.05^a^
Gender (male/female, *n*, %)	1,702/2,445 (41.0/59.0)	1,029/1,332 (43.6/56.4)	<0.05^a^
Education level (years, *n* %)			>0.05^a^
<9	1,808 (43.6)	1,039 (44.0)	
9-	1,411 (34.0)	815 (34.5)	
≥13	928 (22.4)	507 (21.5)	
Marital status (married/others, *n*, %)	3,640/507(87.8/12.2)	2,081/280 (88.1/11.9)	>0.05^a^
Active smoking status (*n*, %)			<0.05^a^
Never	3,365 (81.1)	1,840 (77.9)	
Quit	390 (9.4)	271 (11.5)	
Current	392 (9.5)	250 (10.6)	
Passive smoking status (yes/no, *n*, %)	464/3,683 (11.2/88.8)	265/2,096 (11.2/88.8)	>0.05^a^
Drinking status (*n*, %)			<0.05^a^
Never	3,586 (86.5)	1,970 (83.4)	
Quit	109 (2.6)	59 (2.5)	
Current	452 (10.9)	332 (14.1)	
Hypertension (yes/no, *n*, %)	2,186/1,961 (52.7/47.3)	1,468/893 (62.2/37.8)	<0.05^a^
Diabetes (yes/no, *n*, %)	845/3302 (20.4/79.6)	490/1,871 (20.8/79.2)	<0.05^a^
Hyperlipidemia (yes, no)	3,014/1,133 (72.7/27.3)	1,915/446 (81.1/18.9)	>0.05^a^
BMI (kg/m^2^, *n*, %)			<0.05^a^
<24	2,207 (53.2)	829 (35.1)	
≥24	1,940 (46.8)	1,532 (64.9)	
eGFR (mL/min per 1.73 m^2^, mean ± SD)	82.08 ± 10.5	77.63 ± 10.19	>0.05^b^
Urine creatinine (μmol/L, median, IQRs)	8,399 (4,840, 13,053)	8,209 (4,878, 13,002)	>0.05^c^
**Urinary metal concentration (**μ**g/L, median, IQRs)**			
Lithium	18.78 (10.98, 29.15)	18.24 (10.53, 28.34)	<0.05^c^
Aluminum	24.63 (12.62, 41.37)	24.09 (12.44, 41.10)	>0.05^c^
Titanium	235.10 (136.62, 366.74)	237.97 (134.44, 359.33)	>0.05^c^
Vanadium	2.90 (1.79, 4.15)	2.66 (1.62, 3.76)	<0.05^c^
Chromium	1.57 (0.94, 2.31)	1.50 (0.88, 2.17)	<0.05^c^
Manganese	0.52 (0.26, 0.93)	0.52 (0.26, 0.95)	>0.05^c^
Iron	81.44 (50.90, 123.96)	72.51 (43.07, 110.72)	<0.05^c^
Cobalt	0.21 (0.08, 0.38)	0.20 (0.08, 0.38)	>0.05^c^
Nickel	2.41 (0.08, 0.38)	2.25 (1.23, 3.87)	<0.05^c^
Copper	8.67 (4.84, 13.88)	8.61 (4.68, 13.82)	>0.05^c^
Zinc	374.63 (190.48, 650.51)	408.70 (216.57, 689.45)	<0.05^c^
Arsenic	46.94 (23.21, 90.76)	52.52 (25.59, 101.12)	<0.05^c^
Selenium	31.15 (17.14, 49.94)	31.10 (17.52, 49.83)	>0.05^c^
Rubidium	1,799.86 (1,040.79, 2,785.02)	1,755.76 (1,050.24, 2,687.16)	>0.05^c^
Strontium	90.76 (45.71, 159.16)	82.12 (43.02, 148.68)	<0.05^c^
Molybdenum	47.79 (26.27, 77.69)	45.25 (24.67, 73.65)	<0.05^c^
Cadmium	1.14 (0.56, 2.08)	1.17 (0.57, 2.09)	>0.05^c^
Tin	9.96 (6.32, 15.16)	10.13 (6.40, 15.17)	>0.05^c^
Barium	1.86 (0.99, 3.08)	1.85 (1.01, 3.09)	>0.05^c^
Thallium	0.55 (0.28, 0.91)	0.57 (0.29, 0.91)	>0.05^c^
Lead	1.01 (0.57, 1.64)	0.97 (0.55, 1.66)	>0.05^c^

BMI, body mass index; eGFR, estimated glomerular filtration rate; SD, standard deviation; IQRs, interquartile ranges.

^a^Chi-square test compares the differences in categorical variables' distribution between groups;

^b^Student's *t*-test compares the differences between the two groups of means;

^c^Mann-Whitney U-test is used for the rank sum test of two independent samples.

### Urinary levels of individual metals and HUA risk

**Figure 1:**
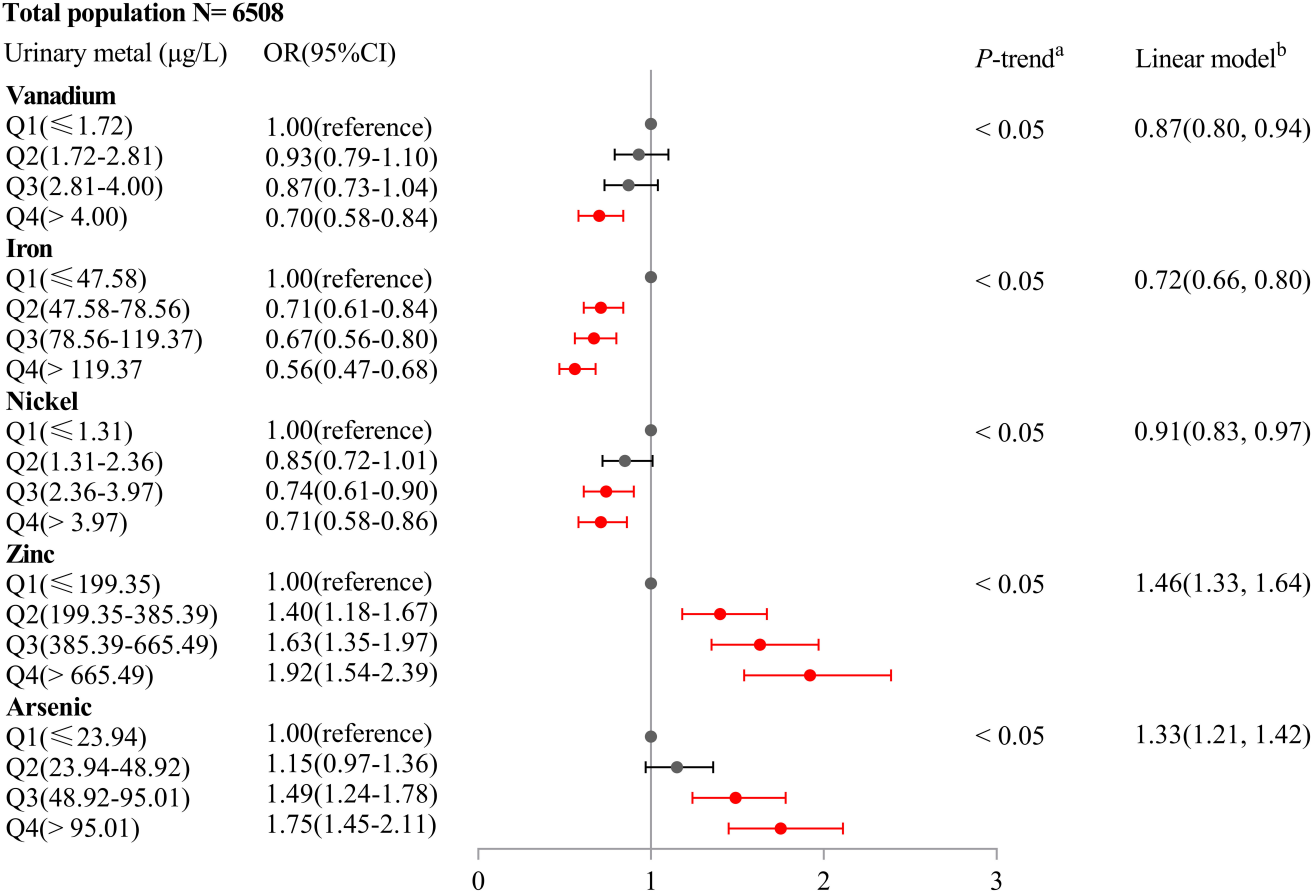
Association of an IQR increase in urinary metal concentrations of vanadium, iron, nickel, zinc, and arsenic with hyperuricemia risk (Odds ratio and 95% confidence interval). Unconditional stepwise logistic regression models were performed and adjusted for age, gender, education level, marital status, active smoking status, passive smoking status, drinking status, hypertension, diabetes, hyperlipidemia, BMI, eGFR, and urine creatinine.

**Figure 2:**
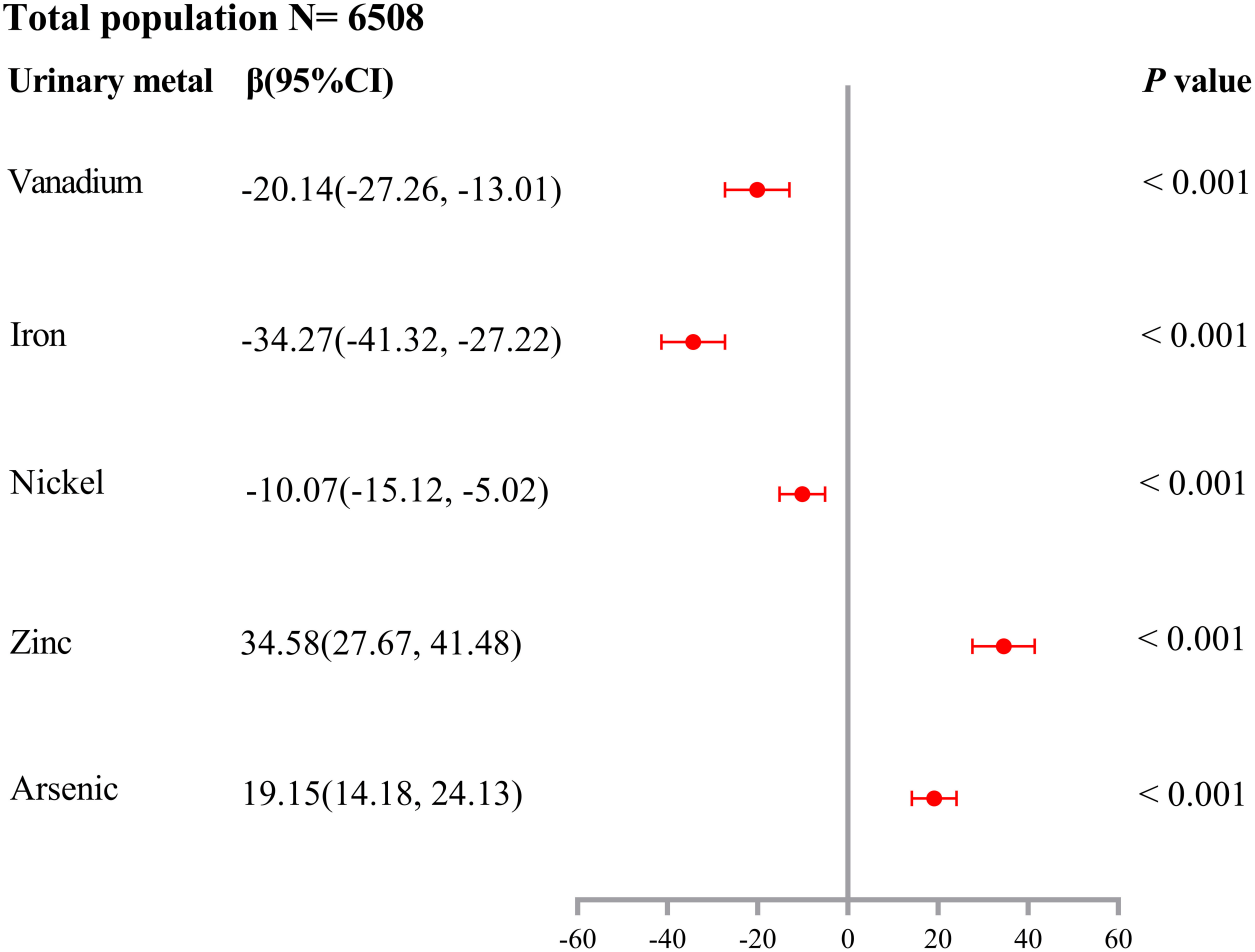
Association of a 1-SD increase in log-transformed urinary metal concentrations of vanadium, iron, nickel, zinc, and arsenic with SUA levels (β and 95% confidence interval). Multiple linear regression models were performed and adjusted for age, gender, education level, marital status, active smoking status, passive smoking status, drinking status, hypertension, diabetes, hyperlipidemia, BMI, eGFR, and urine creatinine.

As shown in Supplementary Table 2, unconditional logistic regression models suggested the association between urinary V (OR = 0.67, 95%CI: 0.57–0.78), Cr (OR = 0.78, 95%CI: 0.66–0.92), Fe (OR = 0.64, 95%CI: 0.55–0.75), Ni (OR = 0.81, 95%CI: 0.68–0.95), Zn (OR = 1.36, 95%CI: 1.14–1.63), and As levels (OR = 1.46, 95%CI: 1.23–1.72) and HUA risk (all *P*_trend_ < 0.05), after adjusting for potential confounders (age, gender, education level, marital status, active smoking status, passive smoking status, drinking status, hypertension, diabetes, hyperlipidemia, BMI, eGFR, and urine creatinine). As shown in Supplementary Figure 2, LASSO regression determined the optimal λ (−4.50) through 10-fold cross-validation based on the minimum MSE. After adjusting for the same confounders in unconditional logistic regression models, urinary metals, V, Fe, Ni, Zn, As, and Mo, were selected as optimal predictors according to LASSO regression models. As shown in Figure 1, we incorporated V, Cr, Fe, Ni, Zn, As, and Mo into the unconditional stepwise logistic regression models, after adjusting for the same confounders in unconditional logistic regression models and found that urinary V (OR = 0.70, 95%CI: 0.58–0.84), Fe (OR = 0.56, 95%CI: 0.47–0.68), and Ni (OR = 0.71, 95%CI: 0.58–0.86) levels were negatively associated with HUA risk (all *P*_trend_ < 0.05), but urinary Zn (OR = 1.92, 95%CI: 1.54–2.39) and As levels (OR = 1.75, 95%CI: 1.45–2.11) were positively associated with HUA risk (all *P*_trend_ < 0.05). As shown in Figure 2, multiple linear regression models showed a lower SUA level of 34.27 (95%CI: −41.32 to −27.22) for a 1-SD increment in log-transformed Fe and a higher SUA level of 34.58 (27.67–41.48) for a 1-SD increment in log-transformed Zn (*P* < 0.001).

As shown in Table 2, we found that Fe was negatively associated with HUA risk in all subgroups (all *P*_trend_ < 0.05). We also found positive associations between the highest quartile (the 75th quartile) of urinary Zn levels and HUA risk in the subgroups of gender, age, BMI, hypertension, diabetes (no), and hyperlipidemia (yes) (all *P*_trend_ < 0.05).

**Table 2:** Subgroup analysis of urinary metal vanadium, iron, nickel, zinc, and arsenic with hyperuricemia risk (OR, 95% CI).

**Subgroup**	**Quartiles of urinary metals levels (**μ**g/L)**				***p*-trend^a^**	***p*-interaction^b^**
	≤**P25**	**P25-**	**P50-**	**P75-**		
**Vanadium**						
Gender						0.278
Male (*n* = 2,731)	1.00 (ref)	0.85 (0.66, 1.10)	0.81 (0.62, 1.07)	0.60 (0.45, 0.80)	0.003	
Female (*n* = 3,777)	1.00 (ref)	1.01 (0.81, 1.25)	0.94 (0.75, 1.18)	0.79 (0.62, 1.01)	0.142	
Age (years)						0.583
<67 (*n* = 3,329)	1.00 (ref)	1.22 (0.96, 1.54)	1.23 (0.88, 1.44)	0.92 (0.71, 1.20)	0.062	
≥ 67 (*n* = 3,179)	1.00 (ref)	0.72 (0.57, 0.90)	0.68 (0.53, 0.87)	0.53 (0.41, 0.70)	0.000	
BMI (kg/m^2)^						0.610
<24 (*n* = 3,036)	1.00 (ref)	1.04 (0.81, 1.34)	0.92 (0.70, 1.20)	0.87 (0.65, 1.17)	0.544	
≥ 24 (*n* = 3,472)	1.00 (ref)	0.85 (0.68, 1.06)	0.82 (0.65, 1.04)	0.59 (0.46, 0.76)	0.000	
Hypertension						0.953
Yes (*n* = 3,654)	1.00 (ref)	1.05 (0.85, 1.30)	0.94 (0.75, 1.18)	0.71 (0.56, 0.91)	0.003	
No (*n* = 2,854)	1.00 (ref)	0.76 (0.59, 0.99)	0.79 (0.59, 1.04)	0.67 (0.50, 0.90)	0.061	
Diabetes						0.272
Yes (*n* = 1,335)	1.00 (ref)	1.01 (0.71, 1.42)	0.86 (0.59, 1.26)	0.67 (0.44, 1.01)	0.142	
No (*n* = 5,173)	1.00 (ref)	0.91 (0.75, 1.09)	0.87 (0.72, 1.07)	0.71 (0.57, 0.87)	0.007	
Hyperlipidemia						0.408
Yes (*n* = 4,929)	1.00 (ref)	1.05 (0.87, 1.26)	0.93 (0.77, 1.14)	0.72 (0.58, 0.89)	0.001	
No (*n* = 1,579)	1.00 (ref)	0.61 (0.43, 0.87)	0.69 (0.47, 0.99)	0.62 (0.42, 0.92)	0.038	
**Iron**						
Gender						0.618
Male (*n* = 2,731)	1.00 (ref)	0.75 (0.60, 0.96)	0.74 (0.57, 0.96)	0.57 (0.43, 0.77)	0.003	
Female (*n* = 3,777)	1.00 (ref)	0.68 (0.54, 0.85)	0.63 (0.50, 0.80)	0.56 (0.44, 0.72)	0.000	
Age (years)						0.544
<67 (*n* = 3,329)	1.00 (ref)	0.62 (0.49, 0.77)	0.56 (0.44, 0.72)	0.53 (0.41, 0.70)	0.000	
≥ 67 (*n* = 3,179)	1.00 (ref)	0.81 (0.64, 1.01)	0.81 (0.63, 1.03)	0.59 (0.45, 0.78)	0.002	
BMI (kg/m^2)^						0.286
<24 (*n* = 3,036)	1.00 (ref)	0.72 (0.57, 0.93)	0.62 (0.47, 0.81)	0.50 (0.38, 0.68)	0.000	
≥ 24 (*n* = 3,472)	1.00 (ref)	0.69 (0.56, 0.85)	0.71 (0.56, 0.89)	0.61 (0.48, 0.79)	0.001	
Hypertension						0.305
Yes (*n* = 3,654)	1.00 (ref)	0.75 (0.61, 0.93)	0.74 (0.60, 0.93)	0.59 (0.46, 0.75)	0.000	
No (*n* = 2,854)	1.00 (ref)	0.66 (0.51, 0.86)	0.59 (0.44, 0.78)	0.53 (0.40, 0.72)	0.000	
Diabetes						0.932
Yes (*n* = 1,335)	1.00 (ref)	0.71 (0.50, 1.01)	0.78 (0.53, 1.14)	0.51 (0.34, 0.78)	0.013	
No (*n* = 5,173)	1.00 (ref)	0.72 (0.60, 0.86)	0.65 (0.54, 0.79)	0.58 (0.47, 0.72)	0.000	
Hyperlipidemia						0.816
Yes (*n* = 4,929)	1.00 (ref)	0.73 (0.61, 0.88)	0.69 (0.57, 0.84)	0.58 (0.47, 0.72)	0.000	
No (*n* = 1,579)	1.00 (ref)	0.64 (0.46, 0.91)	0.65 (0.45, 0.93)	0.54 (0.36, 0.81)	0.017	
**Nickel**						
Gender						0.874
Male (*n* = 2,731)	1.00 (ref)	0.81 (0.62, 1.06)	0.80 (0.59, 1.07)	0.74 (0.54, 1.02)	0.299	
Female (*n* = 3,777)	1.00 (ref)	0.87 (0.70, 1.08)	0.71 (0.56, 0.91)	0.70 (0.54, 0.90)	0.017	
Age (years)						0.594
<67 (*n* = 3,329)	1.00 (ref)	0.67 (0.53, 0.85)	0.62 (0.48, 0.80)	0.67 (0.51, 0.89)	0.001	
≥ 67 (*n* = 3,179)	1.00 (ref)	1.10 (0.86, 1.40)	0.89 (0.68, 1.16)	0.76 (0.57, 1.01)	0.022	
BMI (kg/m^2)^						0.042
<24 (*n* = 3,036)	1.00 (ref)	0.89 (0.69, 1.16)	0.90 (0.68, 1.20)	0.81 (0.60, 1.01)	0.584	
≥ 24 (*n* = 3,472)	1.00 (ref)	0.81 (0.65, 1.02)	0.62 (0.49, 0.79)	0.63 (0.49, 0.82)	0.001	
Hypertension						0.516
Yes (*n* = 3,654)	1.00 (ref)	0.86 (0.69, 1.08)	0.72 (0.56, 0.91)	0.73 (0.56, 0.94)	0.036	
No (*n* = 2,854)	1.00 (ref)	0.82 (0.64, 1.07)	0.76 (0.57, 1.01)	0.68 (0.50, 0.92)	0.092	
Diabetes						0.865
Yes (*n* = 1,335)	1.00 (ref)	0.69 (0.47, 1.01)	0.67 (0.44, 1.01)	0.75 (0.49, 1.14)	0.206	
No (*n* = 5,173)	1.00 (ref)	0.88 (0.73, 1.07)	0.75 (0.61, 0.93)	0.69 (0.55, 0.86)	0.006	
Hyperlipidemia						0.062
Yes (*n* = 4,929)	1.00 (ref)	0.84 (0.70, 1.01)	0.68 (0.55, 0.84)	0.67 (0.53, 0.83)	0.001	
No (*n* = 1,579)	1.00 (ref)	0.90 (0.61, 1.32)	0.91 (0.61, 1.36)	0.87 (0.56, 1.33)	0.926	
**Zinc**						
Gender						0.137
Male (*n* = 2,731)	1.00 (ref)	1.30 (0.95, 1.76)	1.50 (1.08, 2.07)	1.68 (1.17, 2.40)	0.038	
Female (*n* = 3,777)	1.00 (ref)	1.45 (1.17, 1.80)	1.63 (1.28, 2.08)	2.02 (1.52, 2.70)	0.000	
Age (years)						0.725
<67 (*n* = 3,329)	1.00 (ref)	1.30 (1.02, 1.66)	1.52 (1.16, 1.98)	1.92 (1.41, 2.62)	0.001	
≥ 67 (*n* = 3,179)	1.00 (ref)	1.51 (1.18, 1.93)	1.75 (1.33, 2.30)	1.89 (1.38, 2.58)	0.000	
BMI (kg/m^2)^						0.556
<24 (*n* = 3,036)	1.00 (ref)	1.40 (1.07, 1.83)	1.46 (1.08, 1.96)	1.61 (1.14, 2.27)	0.035	
≥ 24 (*n* = 3,472)	1.00 (ref)	1.38 (1.10, 1.73)	1.74 (1.35, 2.24)	2.12 (1.60, 2.83)	0.000	
Hypertension						0.077
Yes (*n* = 3,654)	1.00 (ref)	1.36 (1.08, 1.70)	1.76 (1.38, 2.25)	1.91 (1.44, 2.54)	0.000	
No (*n* = 2,854)	1.00 (ref)	1.47 (1.12, 1.94)	1.45 (1.07, 1.97)	1.93 (1.36, 2.73)	0.003	
Diabetes						0.114
Yes (*n* = 1,335)	1.00 (ref)	1.16 (0.74, 1.81)	1.40 (0.88, 2.22)	1.46 (0.89, 2.39)	0.432	
No (*n* = 5,173)	1.00 (ref)	1.43 (1.19, 1.73)	1.59 (1.29, 1.97)	1.99 (1.56, 2.55)	0.000	
Hyperlipidemia						0.973
Yes (*n* = 4,929)	1.00 (ref)	1.36 (1.13, 1.67)	1.66 (1.34, 2.06)	1.99 (1.56, 2.54)	0.000	
No (*n* = 1,579)	1.00 (ref)	1.41 (0.96, 2.06)	1.40 (0.91, 2.14)	1.59 (0.97, 2.60)	0.267	
**Arsenic**						
Gender						0.741
Male (*n* = 2,731)	1.00 (ref)	1.23 (0.93, 1.61)	1.44 (1.08, 1.91)	1.84 (1.36, 2.49)	0.001	
Female (*n* = 3,777)	1.00 (ref)	1.10 (0.89, 1.38)	1.52 (1.20, 1.93)	1.68 (1.32, 2.16)	0.000	
Age (years)						0.177
<67 (*n* = 3,329)	1.00 (ref)	1.12 (0.88, 1.43)	1.35 (1.04, 1.75)	1.56 (1.20, 2.03)	0.005	
≥ 67 (*n* = 3,179)	1.00 (ref)	1.17 (0.92, 1.49)	1.62 (1.25, 2.08)	2.00 (1.52, 2.63)	0.000	
BMI (kg/m^2)^						0.442
<24 (*n* = 3,036)	1.00 (ref)	1.25 (0.96, 1.63)	1.33 (1.01, 1.76)	1.72 (1.29, 2.31)	0.003	
≥ 24 (*n* = 3,472)	1.00 (ref)	1.10 (0.88, 1.38)	1.67 (1.31, 2.13)	1.82 (1.41, 2.34)	0.000	
Hypertension						0.955
Yes (*n* = 3,654)	1.00 (ref)	1.13 (0.91, 1.41)	1.44 (1.14, 1.82)	1.60 (1.25, 2.05)	0.001	
No (*n* = 2,854)	1.00 (ref)	1.16 (0.88, 1.52)	1.56 (1.18, 2.07)	2.02 (1.50, 2.72)	0.000	
Diabetes						0.340
Yes (*n* = 1,335)	1.00 (ref)	1.17 (0.80, 1.71)	1.60 (1.08, 2.36)	1.53 (1.01, 2.31)	0.072	
No (*n* = 5,173)	1.00 (ref)	1.16 (0.96, 1.41)	1.49 (1.21, 1.82)	1.84 (1.49, 2.29)	0.000	
Hyperlipidemia						0.169
Yes (*n* = 4,929)	1.00 (ref)	1.07 (0.88, 1.29)	1.37 (1.12, 1.68)	1.64 (1.32, 2.02)	0.000	
No (*n* = 1,579)	1.00 (ref)	1.47 (0.99, 2.16)	1.99 (1.33, 2.98)	2.26 (1.47, 2.47)	0.001	

P25: 25th percentile; P50: 50th percentile; P75: 75th percentile. *p*-interaction, *p*-values for the interaction terms. Subgroup analysis was conducted using logistic regression, and adjusted for age, gender, education level, marital status, active smoking status, passive smoking status, drinking status, hypertension, diabetes, hyperlipidemia, BMI, eGFR, and urine creatinine.

^a^p-values for the trend test were obtained from the logistic regression models using the median of each metal quartile (log10-transformed urinary metal concentrations) as a continuous variable.

^b^The interaction was examined by adding an interaction term between a specific metal and the stratification variable as well as the metals and adjusted for age, gender, education level, marital status, active smoking status, passive smoking status, drinking status, hypertension, diabetes, hyperlipidemia, BMI, eGFR, and urine creatinine.

### Dose–response relationship of urinary metals with HUA risk

**Figure 3:**
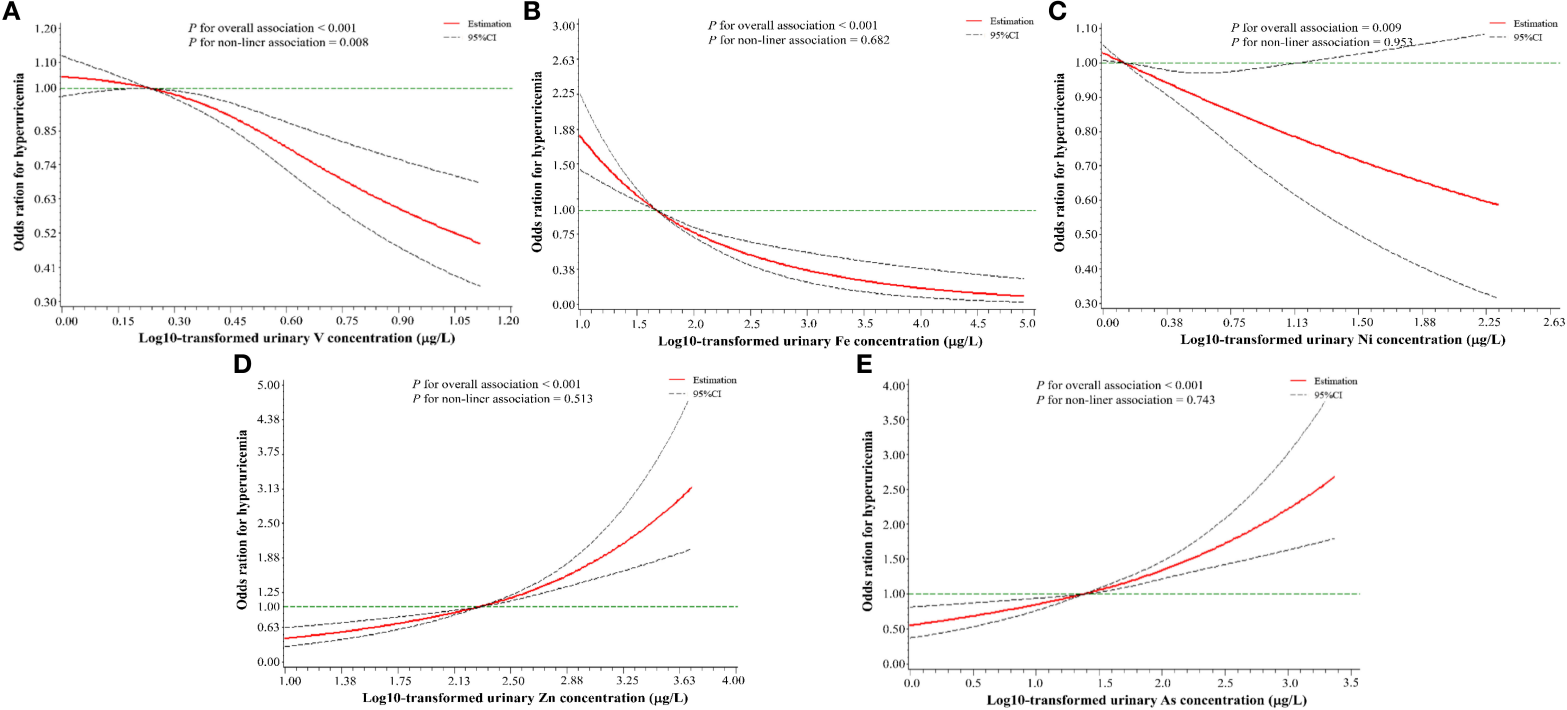
A restricted cubic spline regression model with three knots (the 10th, 50th, and 90th percentile) for urinary metals levels and hyperuricemia risk. **(A)** V (vanadium); **(B)** Fe (iron); **(C)** Ni (nickel); **(D)** Zn (zinc); **(E)** As (arsenic). The X-axis indicates the log10-transformed urinary metal concentrations. Odds ratios (OR) and 95% confidence intervals were estimated, and metal concentrations (log10-transformed) at the 25th percentile were used as the reference value. All of the restricted cubic spline regression models were constructed after adjusting for age, gender, education level, marital status, active smoking status, passive smoking status, drinking status, hypertension, diabetes, hyperlipidemia, BMI, eGFR, and urine creatinine.

As shown in Figure 3, after adjusting for the same confounders in unconditional logistic regression models, RCS logistic regression models showed that there was a negative non-linear dose–response relationship between urinary V levels and HUA risk (*P*_overall_ < 0.001, *P*_nonliner_ = 0.008), but a negative linear dose–response relationship between both urinary Fe (*P*_overall_ < 0.001, *P*_nonliner_ = 0.682) and Ni levels (*P*_overall_ = 0.009, *P*_nonliner_ = 0.953) and HUA risk. A positive linear relationship was found between urinary Zn (*P*_overall_ < 0.001, *P*_nonliner_ = 0.513), or As levels (*P*_overall_ < 0.001, *P*_nonliner_ = 0.743) and HUA risk.

### Effect of additive interaction of Fe and Zn on HUA risk

**Figure 4:**
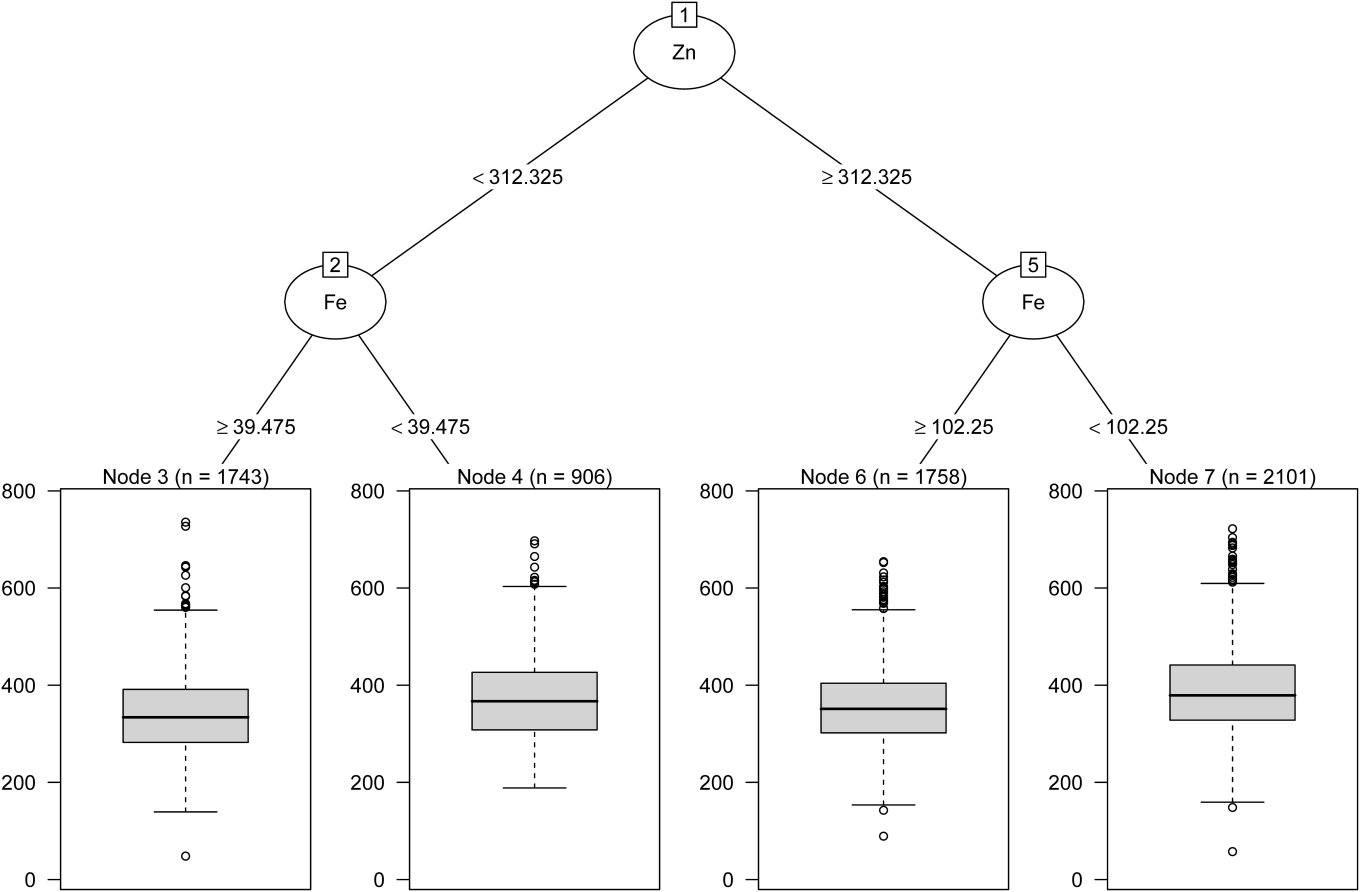
Combined associations between urinary metal levels and SUA concentrations performed with regression tree.

As shown in Table 3, GLM showed an additive interaction between urinary low-Fe (<78.56 μg/L) and high-Zn (≥ 385.39 μg/L) levels on an increased risk of HUA (RERI = 0.31, 95%CI: 0.03–0.59; AP = 0.18, 95%CI: 0.02–0.34; S = 1.76, 95%CI: 1.69–3.49). However, no interaction between the other metals on HUA risk was found. As shown in Figure 4, the regression tree showed urinary Zn levels of ≥ 312.33 μg/L and urinary Fe levels of <102.25 were likely to have the highest concentrations of SUA (Node 7).

**Table 3:** Combined associations of urinary metals with hyperuricemia risk (OR, 95% CI).

**Variable^a^**	**Adjusted OR^b^**	**RERI (95% CI)^c^**	**AP (95% CI)^c^**	**S (95% CI)^c^**
**V-Fe**		−0.26 (−0.57, 0.04)	−0.18 (−0.40, −0.03)	0.62 (0.38, 1.01)
High V + High Fe	1.00 (ref)			
High V + Low Fe	1.33 (1.13, 1.57)			
Low V + High Fe	1.35 (1.15, 1.59)			
Low V + Low Fe	1.42 (1.24, 1.62)			
**V-Ni**		−0.20 (−0.47, 0.07)	−0.15 (−0.35, 0.051)	0.63 (0.37, 1.07)
High V + High Ni	1.00 (ref)			
High V + Low Ni	1.20 (1.03, 1.40)			
Low V + High Ni	1.34 (1.16, 1.56)			
Low V + Low Ni	1.34 (1.18, 1.53)			
**V-Zn**		0.25 (−0.03, 0.52)	0.15 (−0.01, 0.31)	1.59 (0.84, 3.03)
High V + Low Zn	1.00 (ref)			
Low V + Low Zn	1.18 (0.99, 1.40)			
High V + High Zn	1.24 (1.06, 1.45)			
Low V + High Zn	1.67 (1.39, 2.00)			
**V-As**		0.20 (−0.11, 0.50)	0.10 (−0.05, 0.26)	1.29 (0.84, 1.98)
High V + Low As	1.00 (ref)			
Low V + Low As	1.37 (1.17, 1.62)			
High V + High As	1.31 (1.12, 1.54)			
Low V + High As	1.88 (1.58, 2.25)			
**Fe-Ni**		−0.04 (−0.31, 0.23)	−0.03 (−0.22, 0.17)	0.91 (0.47, 1.78)
High Fe + High Ni	1.00 (ref)			
High Fe + Low Ni	1.15 (0.98, 1.36)			
Low Fe + High Ni	1.26 (1.07, 1.48)			
Low Fe + Low Ni	1.37 (1.19, 1.58)			
**Fe-Zn**		0.31 (0.03, 0.59)	0.18 (0.02, 0.34)	1.76 (1.69, 3.49)
High Fe + Low Zn	1.00 (ref)			
Low Fe + Low Zn	1.18 (0.99, 1.40)			
High Fe + High Zn	1.23 (1.04, 1.44)			
Low Fe + High Zn	1.71 (1.41, 2.07)			
**Fe-As**		0.08 (−0.25, 0.41)	0.04 (−0.12, 0.21)	1.09 (0.76, 1.59)
High Fe + Low As	1.00 (ref)			
Low Fe + Low As	1.23 (1.04, 1.44)			
High Fe + High As	1.35 (1.15, 1.58)			
Low Fe + High As	1.39 (1.20, 1.60)			
**Ni-Zn**				
High Ni + Low Zn	1.00 (ref)	0.01 (−0.31, 0.33)	0.01 (−0.19, 0.20)	1.01 (0.62, 1.67)
Low Ni + Low Zn	1.32 (1.10, 1.58)			
High Ni + High Zn	1.31 (1.10, 1.57)			
Low Ni + High Zn	1.64 (1.34, 2.02)			
**Ni-As**		0.14 (−0.17, 0.45)	0.08 (−0.09, 0.25)	1.21 (0.76, 1.92)
High Ni + Low As	1.00 (ref)			
Low Ni + Low As	1.39 (1.17, 1.64)			
High Ni + High As	1.27 (1.07, 1.50)			
Low Ni + High As	1.79 (1.47, 2.15)			
**Zn-As**		0.11 (−0.17, 0.38)	0.07 (−0.11, 0.26)	1.32 (0.59, 2.94)
Low Zn + Low As	1.00 (ref)			
High Zn + Low As	1.26 (1.07, 1.48)			
Low Zn + High As	1.07 (0.91, 1.27)			
High Zn + High As	1.44 (1.24, 1.68)			

V, Vanadium; Fe, Iron; Ni, Nickel; As, Arsenic; Zn, Zinc; RERI, recommended relative excess risk due to interaction; AP, attributable proportion due to interaction; S, synergy index; OR: odd ratios; 95% CI: 95% confidence interval. ^a^Low V <2.81 μg/L, High V ≥ 2.81 μg/L; Low Fe <78.56 μg/L, High Fe ≥ 78.56 μg/L; Low Ni <2.36 μg/L, High Ni ≥ 2.36 μg/L; Low Zn <385.39 μg/L, High Zn ≥ 385.39 μg/L; Low As <48.92 μg/L, High As ≥ 48.92 μg/L.

^b^Generalized linear model was used to analyze combined associations between urinary metals with hyperuricemia risk after adjusted for age, gender, education level, marital status, active smoking status, passive smoking status, drinking status, hypertension, diabetes, hyperlipidemia, BMI, eGFR, and urine creatinine.

^c^When the 95% CI of RERI and AP does not contain 0 and the 95% CI of S does not contain 1, the interaction between the two is considered to be statistically significant.

## Discussion

We found that higher urinary V, Fe, and Ni levels were linked to a lower risk of HUA in addition to the positive association of urinary Zn and As levels with HUA risk. Moreover, the additive interaction between low-iron (<78.56 μg/L) and high-zinc (≥385.39 μg/L) levels greatly increased the risk of HUA in elderly adults.

We also found that the median urinary level of As was 48.92 μg/L, which was 5.9-fold higher than that of individuals aged >20 years (*n* = 5,632) from the NHANES 2003–2010 (14) and 3.2-fold higher than that of individuals aged 44.9–56.0 years (*n* = 1,335) from the Study of Women's Health Across the Nation (32). In addition, median urinary V levels (2.81 μg/L) of the individuals were 1.9-fold higher than that of older adults aged >60 years (*n* = 3,814) in Anhui province, China (33). In addition, median urinary levels of Fe (78.56 μg/L) or Zn (385.39 μg/L) levels were higher than that (*n* = 3,272, 54.67 μg/L for Fe, 310.94 μg/L for Zn) of individuals from the Wuhan-Zhuhai cohort (34). The reasons may be related to gender, age, region, environmental exposure, lifestyle, and population size. In the present study, older adults (aged ≥60 years) who have lived in Shenzhen since the early stage of the city's construction tend to have had a longer exposure to metals in the environment and seafood because Shenzhen is a coastal city in South China.

Previous studies revealed consistent results to support the positive association between blood Fe concentrations and HUA risk. For example, a cross-sectional and longitudinal study in the employees of Zhenhai Refining and Chemical Company, Ningbo, China (*n* = 10,074) (35) indicated that exposure to high serum ferritin (SF) levels was linked to an increased risk of HUA (HR = 1.65, 95%CI: 1.38–1.96) after adjusting for age and gender. Results from the 2009 China Health and Nutrition Study (*n* = 7,946) (36) revealed that individuals with the highest quartile (the 75th quartile: 237.8 μg/L) of SF levels were at higher risk for HUA (OR = 3.09, 95%CI: 2.45–3.89), as compared with those in the lowest quartile (the 25th quartile: 20.3 μg/L). A recent study conducted in Xiangya Hospital, Central South University, Changsha, China (37) reported a link between serum Fe (OR = 1.56, 95%CI: 1.14– 2.13) or SF (OR = 2.25, 95%CI: 1.54–3.29) concentrations and the prevalence of HUA in adults (*n* = 2,824, aged 52.2 ± 7.2 years); however, we found a negative association between urinary Fe levels and HUA risk, which may be due to a difference in biological significances between urinary Fe and blood Fe concentrations. Because urinary Fe concentrations generally represent the levels of Fe in the mucosal cells of the urinary tract and the circulating Fe (transfer Fe protein) (38). When blood Fe concentration is too high, oxidative damage to the renal tubules can be induced, resulting in decreased renal tubular reabsorption of transferrin and UA, increased excretions of UA and urinary iron, and decreased SUA levels (39, 40). A recent study on multiple metals (13 blood metals) exposure and HUA risk from Shenzhen city, China (*n* = 1,406, aged from 31 to 91 years) (15) reported that there was no association between plasma Fe levels and HUA risk (median of plasma Fe level: non-HUA = 1,697.50 μmol/L, HUA = 1,697.48 μmol/L). The reason may be that the interactions of multiple metals may weaken the effect of plasma Fe on HUA. The association between Fe exposure and HUA was related to several factors, including detected concentrations of Fe in different biological samples, regions, and species.

Some controversial results about the relationship between Zn levels (plasma Zn or dietary Zn intake) and HUA risk have been reported in previous studies (15, 41, 42). We found a positive linear dose–response relationship between urinary Zn levels with HUA risk, which is inconsistent with the previous findings that dietary Zn intake was inversely linked to HUA risk (41, 42). In the individuals (*n* = 24,975, aged ≥ 20 years) from the NHANES 2001–2014, dietary Zn intake was found to be inversely correlated with HUA risk (41), the same finding was found in adults (*n* = 5,168, aged ≥ 40 years) from the Department of Health Examination Center, Xiangya Hospital, Changsha, China (42). We note the consistent finding of a positive correlation between Zn exposure and HUA risk after comparing the findings in a multiple-metal exposure study (*n* = 1,406, mean age: 58.89 ± 9.54 years) on plasma Zn (15) and this study; both studies were conducted in older adults in Shenzhen, China. Nevertheless, there were differences in the types of metals (13 metals vs. 21 metals), biological samples (plasma or urinary), and measurement methods for Zn concentrations in the individuals. Lack of zinc will lead to cardiovascular disease, growth restriction, and increased cancer susceptibility (43), while excessive zinc can produce toxic effects (44, 45). We suppose that if there is an appropriate dose of zinc concentrations, deficiency or excess will be caused varying degrees of hazards. The elderly in coastal cities from southern China may have excessive zinc due to environmental exposure and seafood diets. In addition, the interactions of multiple metals may enhance the risk of Zn to HUA. Overall, the relationship between Zn and HUA risk is still inconclusive, and further studies are needed to validate this finding.

Subgroup analysis suggested that individuals with BMI (≥24), hyperlipidemia (yes), and urinary Zn (≥385.39 μg/L) were at higher risk of HUA. The reasons for this may be that Zn transporters are differentially expressed in various tissues of the body, and obesity and other diseases can increase the accumulation of Zn in adipose tissue (one of the most important Zn sources) and can reduce the Zn concentration in blood (46). A recent study at Yaroslavl State University, Yaroslavl, Russia (*n* = 395, aged from 20 to 60 years) (47) indicated that urine Zn levels in obese individuals (*n* = 196) were 18% higher than that in lean individuals (*n* = 199). In obese individuals, the internal balance of plasma Zn and the process of muscle metabolism may be changed, leading to increased excretion of Zn by urine (48).

We revealed the additive interaction between urinary low-Fe (<78.56 μg/L) and high-Zn (≥385.39 μg/L) levels on the risk of HUA, but previous studies on this were very limited. The interaction between Fe and Zn in the human body absorption process has been widely studied, but the results were inconsistent. Previous studies suggested that the absorptions of Fe and Zn exhibit a competitive inhibition (49, 50). Solomons and Jacob performed a study on assessing iron–zinc interaction with the increasing proportion of Fe and Zn in cola beverage (the ratios of Fe:Zn were 1:1, 2:1, and 3:1) (49), and the results showed that plasma Zn concentrations were decreased. A community-based randomized controlled trial in Indonesia explored the interactions between Fe and Zn in infants (50), and they found that the combination of iron–zinc supplements was not as effective as a single supplement. Additional studies have found a positive interaction between Fe and Zn in human absorption (51–53). A 6-month randomized, double-blind trial investigated the effect of Zn supplementation on the biochemical status of Fe in individuals aged 55–75 (*n* = 188) and 70–85 (*n* = 199) years old (51), suggesting that 15 or 30 mg/d Zn supplementation significantly increased serum Zn levels and urinary Zn excretion, but had no effect on Fe status. A randomized single-blind placebo-controlled trial of pregnant women in the United Kingdom (52) indicated that dietary Fe supplementation (100 mg Fe/d) had no detectable adverse effect on Zn metabolism and increased Zn absorption efficiency in late pregnancy. A randomized controlled trial in Bangladesh reported that the combination of Fe and Zn had the same effect as single administration on reducing diarrhea, hospitalization, or improving Fe status (53). Animal experiments indicated that the interactions between Fe and Zn may depend on their ratios. For instance, no significant inhibition of Zn absorption was found in the digestion and absorption of Zn sulfate (100 μmol/L) in rats (ranging from 0 to 1,000 μmol Fe/L) in the presence of Fe gluconate when the ratio of Fe to Zn was <2:1, and dose-dependent inhibition of Zn absorption between 2:1 and 5:1 reached a plateau beyond this ratio (54). Based on urinary Zn levels, we have two hypotheses. One is Zn deficiency (55): the absorption of Fe in the body also inhibits the absorption of Zn, and the content of Zn excreted in urine increases. In addition, the study population was over 60 years old, and the reduction of Fe accumulation and Zn absorption due to aging may be associated with HUA risk (56). The other hypothesis is that there was excess Zn (57). The study population is located in the coastal areas, the intake of zinc-rich seafood is higher, and the antagonism of the Fe and Zn interaction is much smaller with the increased Zn intake. However, the current research on the iron–zinc interaction and the risk of HUA is very limited, further mechanistic studies are needed regarding the effect of iron-zinc interaction on HUA risk.

There are several strengths in this study. First, we explored the association between urinary multi-metal levels and HUA risk in a large sample size (*n* = 6,508) after adjusting for the traditional confounding factors such as gender, age, BMI, hypertension, and diabetes. Second, we used both LASSO regression and logistics regression for metal selection and RCS logistic regression and GLM for assessing the dose–response relationship and interactions between urinary metals and HUA risk. However, there are still some limitations in this study. First, we did not assess the dietary exposure of individuals, whereas dietary intake is an important factor related to metal exposure in the body. Second, we only detected urinary metal concentrations, which is a limitation in assessing metal exposure of the body, because day-to-day variability of urinary metals concentrations and creatinine excretion of the body can result in measurement errors. But, it cannot deny the role of urinary sample, because of the high amount of metal excretion *via* urine, and non-invasive and convenience in collection of urine sample. Finally, the cross-sectional study is unable to identify the causal relationship between multiple metals exposure levels of the body and HUA. Further prospective studies are needed to validate the findings.

## Conclusion

We found a negative association between higher levels of urinary V, Fe, and Ni and HUA risk and a positive association between urinary Zn and As and HUA risk. Additive interaction of low-Fe (<78.56 μg/L) and high-Zn (≥385.39 μg/L) levels are related to a higher risk of HUA. The findings indicated the potential importance of Zn and Fe exposure in the body in the prevention of elevated SUA levels and HUA risk.

## Data availability statement

The raw data supporting the conclusions of this article will be made available by the authors, without undue reservation.

## Ethics statement

The studies involving human participants were reviewed and approved by the research protocol was approved by the Medical Ethics Research Committee of Shenzhen Center for Disease Control and Prevention (approval numbers: R2017001 and R2018020). The patients/participants provided their written informed consent to participate in this study.

## Author contributions

CH and EG: conceptualization and methodology. CH, EG, JL, WL, and YL: formal analysis. XR and QW: data curation. CH, EG, and FX: writing—original draft preparation. CH and JL: writing—review and editing. DW and QS: supervision. XC and KH: project administration. HH and JL: funding acquisition. All authors have read and agreed to the published version of the manuscript.
