# Supplementary material for: The relative and interactive effects of urinary multiple metals exposure on hyperuricemia among urban elderly in China
Source: Front Public Health. 2023 Feb 13;11:1015202. doi: 10.3389/fpubh.2023.1015202 (PMC9969194; doi:10.3389/fpubh.2023.1015202)

The relative and interactive effects of urinary multiple metals exposure on hyperuricemia among urban elderly in China: Supplementary Materials

Chao Huang [1, †], Erwei Gao [1, †], Feng Xiao [2], Qiongzhen Wu [2], Wei Liu [1], Yi Luo [3], Xiaohu Ren [1], Xiao Chen [1], Kaiwu He [1], Haiyan Huang [1], Qian Sun [1], Desheng Wu [1], Jianjun Liu [1*]

1 Key Laboratory of Modern Toxicology of Shenzhen, Shenzhen Medical Key Discipline of Health Toxicology
(2020-2024), Shenzhen Center for Disease Control and Prevention, Longyuan Road 8, Nanshan District, Shenzhen, 518055, Guangdong, China

2 Food Inspection and Quarantine Technology Center of Shenzhen Customs District, Shenzhen, 518045, Guangdong, China

3 Shenzhen Luohu Hospital for Traditional Chinese Medicine, Shenzhen Luohu Hospital Group, Shenzhen, 51800, Guangdong, China

[†] These authors contributed equally to this work.

***Corresponding author**: Professor Jianjun Liu, junii8@126.com

Running head: multiple metals exposure and hyperuricemia

Table S1 Limits of detection, limits of quantification, detection rate of metal concentrations and Spike recoveries in urine.

Urinary metals	LOD (μg/L)	LOQ (μg/L)	LOQ/2 (μg/L)	N (%)<LOQ	Spike recoveries (%)		
					Seronom L-1	Seronom L-2	SRM1640a
Lithium	0.05	0.15	0.08	0.06	97.14	84.97	105.39
Beryllium	0.01	0.05	0.02	100.00	93.70	83.33	107.56
Aluminum	0.41	1.37	0.69	5.54	110.86	100.43	111.77
Titanium	0.63	2.09	1.05	0.78	152.86	116.56	105.63
Vanadium	0.02	0.07	0.04	0.70	108.35	100.02	105.69
Chromium	0.04	0.14	0.07	4.75	100.76	93.69	105.84
Manganese	0.02	0.08	0.04	10.32	103.04	95.98	105.80
Iron	1.66	5.54	2.77	1.25	103.96	98.45	106.13
Cobalt	0.01	0.03	0.01	15.03	99.89	92.10	104.80
Nickel	0.02	0.08	0.04	2.48	98.32	88.74	106.20
Copper	0.08	0.26	0.13	4.36	90.40	81.29	105.61
Zinc	0.13	0.44	0.22	0.08	110.68	66.04	108.52
Arsenic	0.04	0.13	0.07	0.04	116.11	104.60	109.28
Sselenium	0.33	1.11	0.55	0.75	83.08	75.26	78.47
Rubidium	0.02	0.07	0.03	0.65	111.98	94.72	105.45
Strontium	0.03	0.09	0.04	0.17	110.85	103.45	105.62
Molybdenum	0.06	0.21	0.11	0.45	111.12	104.89	107.45
Cadmium	0.01	0.03	0.02	1.76	109.23	97.91	108.65
Indium	0.00	0.01	0.01	69.52	107.47	99.62	105.21
Tin	0.08	0.26	0.13	1.22	107.18	100.94	104.85
Antimony	0.03	0.11	0.05	22.33	114.75	104.46	108.09
Barium	0.01	0.02	0.01	2.50	111.28	102.28	107.42
Thallium	0.00	0.01	0.00	5.29	105.42	99.35	108.25

Lead	0.01	0.02	0.01	3.09	105.39	97.79	109.50

Note: LOD: limits of detection; LOQ: limits of quantification.

Table S2 Association of an IQR increase in 21 urinary metals concentrations with hyperuricemia risk (OR, 95%CI).

Metals	Quartiles of urinary metals levels (μg/L)				*p*-trend[a]	Linear model[b]
	≤P25	P25-	P50-	P75-		
Lithium	1	0.95 (0.82, 1.11)	0.99 (0.85, 1.16)	0.96 (0.82, 1.16)	> 0.05	0.94 (0.89, 1.01)
Aluminum	1	0.95 (0.82, 1.11)	0.99 (0.85, 1.15)	1.02 (0.88, 1.19)	> 0.05	0.99 (0.95, 1.04)
Titanium	1	0.91 (0.79, 1.06)	1.05 (0.90, 1.23)	0.99 (0.83, 1.18)	> 0.05	1.00 (0.94, 1.06)
Vanadium	1	0.94 (0.81, 1.09)	0.87 (0.75, 1.01)	0.67 (0.57, 0.78)	< 0.05	0.88 (0.83, 0.93)
Chromium	1	0.94 (0.81, 1.10)	0.91 (0.78, 1.06)	0.78 (0.66, 0.92)	< 0.05	0.92 (0.87, 0.97)
Manganese	1	0.99 (0.85, 1.15)	0.99 (0.85, 1.15)	1.05 (0.91, 1.22)	> 0.05	1.01 (0.95, 1.07)
Iron	1	0.78 (0.67, 0.90)	0.75 (0.65, 0.88)	0.64 (0.55, 0.75)	< 0.05	0.84 (0.79, 0.90)
Cobalt	1	0.94 (0.81, 1.09)	0.89 (0.76, 1.04)	0.92 (0.78, 1.08)	> 0.05	0.99 (0.93, 1.06)
Nickel	1	0.91 (0.78, 1.06)	0.80 (0.68, 0.93)	0.81 (0.68, 0.95)	< 0.05	0.92 (0.86, 0.97)
Copper	1	0.91 (0.78, 1.06)	0.92 (0.79, 1.07)	0.91 (0.77, 1.08)	> 0.05	0.96 (0.91, 1.01)
Zinc	1	1.14 (0.98, 1.33)	1.22 (1.04, 1.43)	1.36 (1.14, 1.63)	< 0.05	1.14 (1.05, 1.23)
Arsenic	1	1.01 (0.87, 1.18)	1.24 (1.06, 1.46)	1.46 (1.23, 1.72)	< 0.05	1.17 (1.09, 1.26)
Selenium	1	1.07 (0.92, 1.25)	1.09 (0.93, 1.28)	1.09 (0.91, 1.31)	> 0.05	1.00 (0.93, 1.08)
Rubidium	1	1.09 (0.94, 1.26)	1.07 (0.91, 1.25)	0.95 (0.80, 1.13)	> 0.05	0.98 (0.94, 1.02)
Strontium	1	0.97 (0.83, 1.12)	0.89 (0.77, 1.04)	0.85 (0.72, 1.00)	> 0.05	0.93 (0.87, 1.00)
Molybdenum	1	0.95 (0.82, 1.10)	0.91 (0.78, 1.07)	0.84 (0.70, 1.00)	> 0.05	0.93 (0.87, 1.00)

Cadmium	1	1.03 (0.89, 1.20)	1.13 (0.96, 1.32)	1.17 (0.99, 1.40)	> 0.05	1.05 (0.97, 1.12)
Tin	1	0.98 (0.84, 1.14)	1.06 (0.91, 1.23)	1.02 (0.87, 1.19)	> 0.05	0.99 (0.94, 1.04)
Barium	1	1.03 (0.88, 1.19)	0.96 (0.83, 1.12)	0.96 (0.82, 1.11)	> 0.05	0.98 (0.93, 1.03)
Thallium	1	0.98 (0.84, 1.14)	1.17 (1.00, 1.36)	1.05 (0.89, 1.24)	> 0.05	1.01 (0.96, 1.06)
Lead	1	1.00 (0.86, 1.17)	0.89 (0.76, 1.04)	1.01 (0.86, 1.19)	> 0.05	0.99 (0.94, 1.04)

P25: 25th percentile; P50: 50th percentile; P75: 75th percentile; IQR, interquartile range; OR: odd ratios; 95% CI: 95% confidence interval. Unconditional logistic regression models were performed to assess associations between 21 metals and hyperuricemia risk after adjusted for age, gender, education level, marital status, active smoking status, passive smoking status, drinking status, hypertension, diabetes, hyperlipidemia, BMI, eGFR and urine creatinine.

[a] *p*-values for trend test were obtained from the logistic regression models using the median of each metal quartile (Log10-transformed urinary metal concentration) as a continuous variable.

[b] Urinary metal concentrations were included in the linear model as continuous variables which Log10-transformed and then divided by the corresponding interquartile range (Log10-transformed metal concentrations), and adjusted for age, gender, education level, marital status, active smoking status, passive smoking status, drinking status, hypertension, diabetes, hyperlipidemia, BMI, eGFR and urine creatinine.

Figure S1. Heatmap of the Spearman's correlations coefficients among urinary concentrations 21 metals. The values are shown as different degree of color intensity (red, positive correlation), darker color represents stronger correlation. Abbreviations: lithium, Li; aluminum, Al; titanium, Ti; vanadium, V; Chromium, Cr; manganese, Mn; iron, Fe; cobalt, Co; nickel, Ni; copper, Cu; zinc, Zn; arsenic, As; selenium, Se; rubidium, Rb; strontium, Sr; molybdenum, Mo; Cadmium, Cd; tin, Sn; barium, Ba; thallium, Tl; lead, Pb.

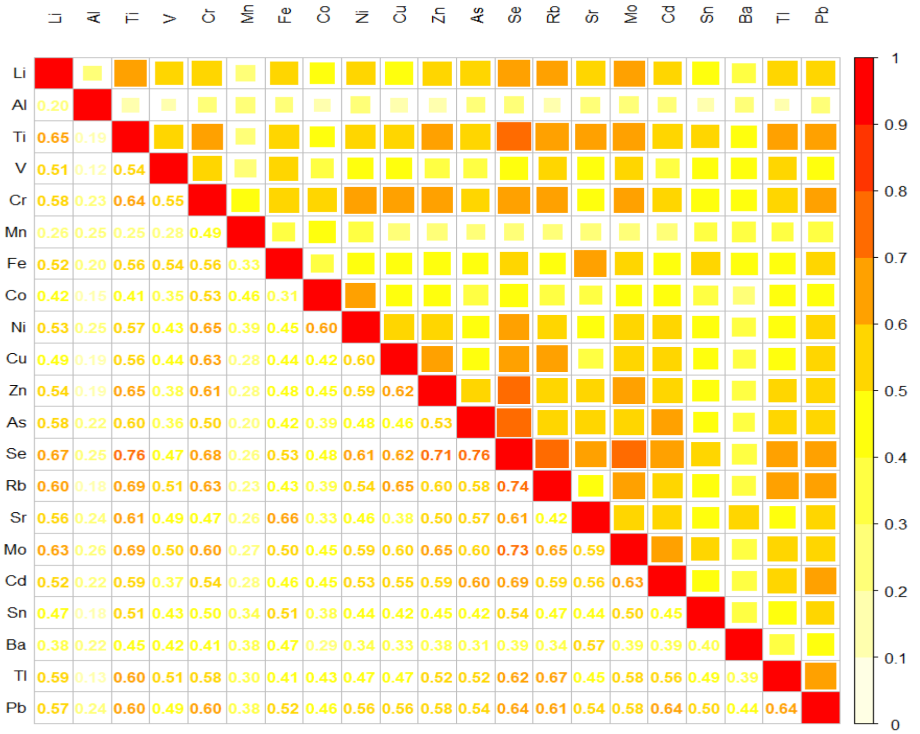

Figure S2. The least absolute shrinkage and selection operator penalized logistic regression for the associations between urinary concentrations of multiple metals and hyperuricemia risk. (a) for 10-fold cross validation; (b) represented coefficient profiles for urinary metals. V(vanadium), Fe (iron), Ni (nickel), Zn (zinc), As (arsenic) and Mo (molybdenum) were selected at the lambda indicated by the dashed red line (at the note of the largest lambda, MSE within one standard error of the minimum MSE was given).

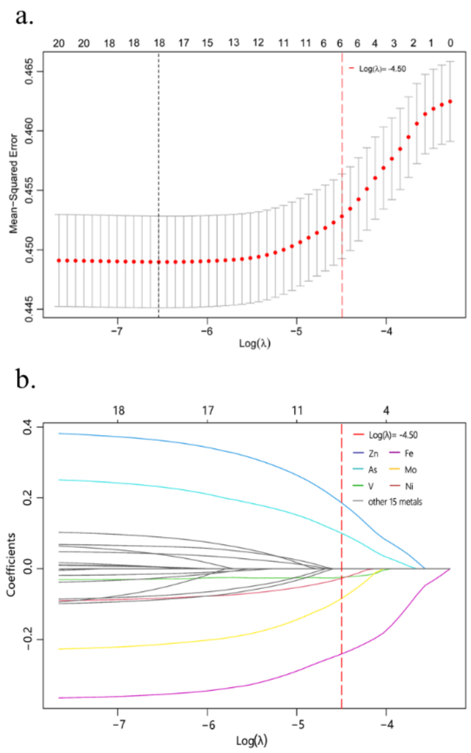

Supplement: Supplementary file 1 [file Data_Sheet_1.pdf]
